# The IJHPR celebrates five years of quality publication

**DOI:** 10.1186/s13584-016-0126-z

**Published:** 2016-12-28

**Authors:** Bruce Rosen, Avi Israeli

**Affiliations:** 1 Smokler Center for Health Policy Research, Myers-JDC-Brookdale Institute, JDC Hill, POB 3886, Jerusalem, 91130 Israel; 2 Hadassah – Hebrew University Medical Center, Jerusalem, Israel; 3 Ministry of Health, Jerusalem, Israel

## Abstract

The Israel Journal of Health Policy Research (IJHPR) will soon be completing its fifth year of publication. In the 5 years since the IJHPR’s launch in January 2012, it has published over 250 articles, achieved an impact factor of 1.35, and secured a place in many of the leading journal databases including, most recently, Medline. The IJHPR’s annual rate of submissions continues to increase, with more and more submissions focusing on major system-wide developments in Israeli health care.

The journal’s accomplishments reflect the hard work and significant contributions of its authors, reviewers, commentators, editorial board members and sponsor - the National Institute for Health Policy Research.

New initiatives for 2017 include symposia highlighting the implications for Israeli health policy of selected IJHPR articles and a collection of essays by editors of leading health policy journals about the opportunities and challenges currently facing journals in our field.

In parallel with the growth and maturation of the IJHPR, there have also been important advances in the volume, quality and visibility of Israeli health services and health policy research more generally. The number of Israeli health care professionals engaged in writing and reviewing manuscripts has increased significantly, and there is growing interest among international journals in manuscripts about Israeli health care. We are confident that in the years ahead we will continue to witness significant achievements for both the journal and for the Israeli health policy and health services research community.

The Israel Journal of Health Policy Research (IJHPR) will soon be completing its fifth year of publication. In the 5 years since the IJHPR’s launch in January 2012, it has published over 250 articles, achieved an impact factor of 1.35, and secured a permanent place in many of the leading journal databases including the Social Science Citation Index, the Web of Science, Scopus, PubMed and – 2 months ago - Medline.

Medline is recognized as the most important and most comprehensive bibliographic resource in the field of biomedicine, and it is managed by the largest medical library in the world - the National Library of Medicine (NLM) [1, 2]. It is also one the world’s most selective databases of peer-reviewed journals.

The IJHPR has been included in a related NLM database – PubMed – since mid-2012. The recent acceptance into Medline is an indication of the journal’s progress in terms of quality, impact and reputation. Acceptance into Medline should also help to increase the journal’s citation rate (though not immediately), as Medline is one of the main search engines used in systematic reviews. The indexing of IJHPR in Medline will begin with volume 5 (2016).

Another important recent achievement is that we are receiving more manuscripts about major system-wide developments in the Israeli health care arena. The topics covered include: the national initiative to increase health care equity [3], the pediatric dental reform [4], medical education [5, 6], advanced practice providers [7], tobacco control [8], and growing pharmaceutical expenses [9, 10]. In addition, since our last editorial [11] (December 2014), the IJHPR has published its first article by a Nobel Prize laureate [12], and its first contributions from China [13] and India [14].

The IJHPR’s rate of submissions has risen substantially since the journal’s launch in 2012, with over 100 manuscripts submitted over the course of 2016. In conjunction with the increase in submissions, we have been steadily raising the journal’s quality bar.

The journal’s accomplishments are due, first and foremost, to the many authors who choose to submit their manuscripts to the IJHPR and, secondly, to the highly professional reviewers whose detailed comments have often made a major contribution to the quality of the published articles. Almost all of Israel’s universities and research centers are represented among the IJHPR’s authors, and many professionals based at Israel’s Ministry of Health, health plans, and hospitals have also published in the IJHPR. The authors’ fields of expertise, and their IJHPR articles, span a very broad range of aspects of health policy – just as we had hoped when we launched the journal 5 years ago [15]. The journal also continues to draw in commentaries from internationally renowned scholars from all around the world. Their commentaries underscore the international and universal significance of the Israeli studies and bring the experience of other countries to bear on the challenges facing Israeli health care.

In addition, the IJHPR considers and publishes research papers initiated by authors outside Israel, so long as they address a subject or contain information relevant to health services and health policy in Israel. The number of such submissions and publications is also increasing, contributing to the IJHPR’s being both an Israeli and an international journal.

The journal could not exist without the ongoing support of its sponsor, the National Institute for Health Policy Research. In addition to material support, the National Institute and its leadership provides the journal with important substantive input, advice and guidance.

We are also profoundly thankful to the members of the editorial board for their input on journal policy and emphases as well as sharing in the nitty gritty work of reviewing manuscripts and identifying additional reviewers.

We are planning to launch two new initiatives over the coming year. First, in honor of the IJHPR’s fifth anniversary we have invited the editors of several leading health policy and health services journals to write essays about the opportunities and challenges currently facing journals in our field. We hope to publish these essays as a special collection.

Second, in conjunction with the leadership of the National Institute for Health Policy Research, we will be experimenting with high-level symposia which will explore the policy implications of one or more IJHPR articles on a pressing policy issue. The first such symposium will be on the growing expenditures on cancer medications and the program will include presentations by authors of the relevant IJHPR articles [9, 10, 16, 17], along with presentations from Israeli health care leaders who are grappling with this issue.

In conclusion, we are pleased to share with you several important developments regarding the publication of articles about Israeli health care (that go well beyond those articles published in the IJHPR).

- The number of health policy articles (based on a keyword search of the Web of Science) with at least one Israeli-based author increased by over 80% between 2007–2011 and 2012–2016.
- Editors of several leading international health and health policy journals have expressed interest in publishing more articles about Israel in the years ahead, and various concrete outreach efforts are already underway.
- A recent study commissioned by Israel’s National Institute for Health Policy Research found that approximately 40% of the studies that they had funded have generated at least one article in a peer-reviewed journal. This figure is higher than many of us expected, and chances are that – now that this measure is being monitored and publicized - it will increase in the years ahead.

In short, the number of articles about Israeli health care is growing and can be expected to grow further in the future. We at the IJHPR are proud to be playing our small part in this. The growth is beneficial not only for Israeli researchers and all the journals involved; it is also extremely important for progress in health care in Israel and beyond. We are confident that the years ahead will also be years of significant achievements for both the journal and for the Israeli health policy/health services research community.

## Abbreviations

IJHPR: Israel Journal of Health Policy Research
NLM: National Library of Medicine
